# Dissection of Anti-tumor Activity of Histone Deacetylase Inhibitor SAHA in Nasopharyngeal Carcinoma Cells *via* Quantitative Phosphoproteomics

**DOI:** 10.3389/fcell.2020.577784

**Published:** 2020-11-26

**Authors:** Huichao Huang, Ying Fu, Ye Zhang, Fang Peng, Miaolong Lu, Yilu Feng, Lin Chen, Zhuchu Chen, Maoyu Li, Yongheng Chen

**Affiliations:** ^1^Department of Oncology, NHC Key Laboratory of Cancer Proteomics, XiangYa Hospital, Central South University, Changsha, China; ^2^Molecular and Computational Biology Program, Department of Biological Sciences, University of Southern California, Los Angeles, CA, United States; ^3^Department of Chemistry, University of Southern California, Los Angeles, CA, United States; ^4^Department of Gastroenterology, XiangYa Hospital, Central South University, Changsha, China; ^5^National Clinical Research Center for Geriatric Disorders, XiangYa Hospital, Central South University, Changsha, China

**Keywords:** histone deacetylase, suberoylanilide hydroxamic acid, p53–Rb1 signaling pathway, nasopharyngeal carcinoma, quantitative phosphoproteomic

## Abstract

Suberoylanilide hydroxamic acid (SAHA), a pan HDAC inhibitor, has been approved by the Food and Drug Administration (FDA) to treat cutaneous T cell lymphoma (CTCL). Nevertheless, the mechanisms underlying the therapeutic effects of SAHA on tumors are yet not fully understood. Protein phosphorylation is one of the most important means to regulate key biological processes (BPs), such as cell division, growth, migration, differentiation, and intercellular communication. Thus, investigation on the impacts of SAHA treatment on global cellular phosphorylation covering major signaling pathways deepens our understanding on its anti-tumor mechanisms. Here we comprehensively identified and quantified protein phosphorylation for the first time in nasopharyngeal carcinoma (NPC) cells upon SAHA treatment by combining tandem mass tags (TMTs)-based quantitative proteomics and titanium dioxide (TiO_2_)-based phosphopeptide enrichment. In total, 7,430 phosphorylation sites on 2,456 phosphoproteins were identified in the NPC cell line 5-8F, of which 1,176 phosphorylation sites on 528 phosphoproteins were significantly elevated upon SAHA treatment. Gene ontology (GO) analysis showed that SAHA influenced several BPs, including mRNA/DNA processing and cell cycle. Furthermore, signaling pathway analysis and immunoblotting demonstrated that SAHA activated tumor suppressors like p53 and Rb1 *via* phosphorylation and promoted cell apoptosis in NPC cells but inactivated energetic pathways such as AMPK signaling. Overall, our study indicated that SAHA exerted anti-tumor roles in NPC cells, which may serve as novel therapeutic for NPC patients.

## Introduction

Nasopharyngeal carcinoma, as a common type of head and neck cancer, has a much higher incidence in Southern China and Southeast Asia, where the annual incidence is about 30 cases per 100,000 persons, in contrast to fewer than one case per 100,000 persons in the United States and Europe (Chang and Adami, 2006; Cao et al., 2011). EBV infection, genetic susceptibility, and environmental factors are involved in the development of NPC (Adham et al., 2012). Although progress has been made in the understanding, diagnosis, and treatment of NPC, it remains to be a serious threat to the community, urging for the development of early diagnostic biomarkers and novel therapeutics.

Histone deacetylases play key roles in regulating chromatin remodeling, the gene expressions of which they regulate epigenetically by turning down histone lysine acetylation in various pathophysiological conditions (Shahbazian and Grunstein, 2007). Both HDACs and histone lysine acetylation are involved in tumorigenesis, and inhibition of HDACs by specific inhibitors has emerged as an effective anti-tumor strategy (Wagner et al., 2010). SAHA (vorinostat), as an inhibitor of HDACs, has been approved by the FDA to treat refractory CTCL (Duvic et al., 2007; Marks and Breslow, 2007; Olsen et al., 2007). Besides CTCL, further research has demonstrated that SAHA processes anti-tumor effects in multiple types of solid tumors, including lung cancer, breast cancer, ovarian cancer, prostate cancer, neuroblastoma, as well as head and neck tumors (Munster et al., 2001; Komatsu et al., 2006; Konstantinopoulos et al., 2014; Xu et al., 2014; Wu et al., 2015). However, the anti-tumor role of SAHA has not been reported in NPC cells. In addition, previous studies merely focused on the alteration of protein acetylation to explain the anti-tumor mechanisms of SAHA.

Protein posttranslational modifications play critical roles in regulating fundamental biological functions, such as gene expression, signal transduction, and cell proliferation, division, and death (Blixt et al., 2004; Krueger and Srivastava, 2006; Hoffman et al., 2008). Prevalent PTMs include phosphorylation, acetylation, methylation, glycosylation, and ubiquitinylation. It has been well established that many PTMs contribute to abnormal cell proliferation, cell adhesion, and morphological changes during cancer progression (Sheehan et al., 2005; Krueger and Srivastava, 2006). Studies have shown that the dysregulated phosphorylation of some cellular signaling pathways contributes to oncogenesis, including receptor tyrosine kinases/PI3-kinase/Akt/mTOR, receptor tyrosine kinases/Ras/Raf/MEK/ERK, MEKK/MKK/JNK, and JAK/STAT (Shawver et al., 2002; Roberts and Der, 2007; Cicenas, 2008; Du et al., 2010). Meanwhile, phosphorylation and dephosphorylation are the key steps to regulate the activities of tumor suppressor genes p53 and Rb1, with the aberrant phosphorylation of both being strongly associated with cancer development, including NPC, breast cancer, lung cancer, and prostate cancer (Bischoff et al., 1990; Gubern et al., 2016; Sanidas et al., 2019). Therefore, the analysis of cancer phosphoproteome is crucial both for providing information on cancer cell signaling and for establishing the basis for targeted therapies.

Liquid chromatography–tandem mass spectrometry is the key technique to characterize and quantify phosphorylated sites and phosphorylated proteins. Due to the low abundance of phosphopeptides in the human body, there are four main strategies to enrich phosphopeptides: IMAC, TiO_2_ affinity chromatography, strong cation exchange, and anti-phosphotyrosine antibodies. Currently, tandem mass tags (TMT)-based quantitative proteomics and TiO_2_-based phosphopeptide enrichment coupled with LC–MS/MS are effective methods to detect, identify, and quantify phosphorylated sites and phosphorylated proteins.

In this study, we hypothesize that SAHA-mediated HDAC inhibition triggers globally reprogrammed signaling events *via* modulating the expression of signaling molecules, including kinases. Subsequently, protein phosphorylation alterations contribute to the inhibitory effect of SAHA on cancers. Combining quantitative phosphoproteomics and bioinformatic analysis, we demonstrated that SAHA treatment altered phosphorylation in key signaling pathways in NPC cells. Meanwhile, we indicated that SAHA inhibits proliferation and induces apoptosis in NPC cells. More importantly, our findings may provide a novel effective therapy for NPC patients and present a useful resource for future studies investigating the in-depth molecular mechanisms underlying the anti-tumor function of SAHA.

## Materials and Methods

### Cell Culture and CCK-8 Assay

The cells were all purchased from American Type Culture Collection and cultured as previously described. 5-8F, HNE3, and 6-10B cells were cultured in RPMI-1640 medium containing 10% fetal bovine serum and 1% penicillin/streptomycin in a humidified environment at 37°C and 5% CO_2_. Cell proliferation and cytotoxicity assay were performed by using CCK-8 (Dojindo, Kumamoto, Japan) according to the manufacturer’s instructions. Briefly, NPC cells were seeded in 96-well plates in triplicate at an initial density of 5 × 10^3^ cells/well. An increased concentration of SAHA (Sigma, St. Louis, MO, United States) was then added to each well for the indicated time. Subsequently, 10 μl of CCK-8 assay solution was added to each well, and the mixture was incubated for 2 h. The absorbance was measured at 450 nm using a multi-well spectrophotometer. Stock solutions of SAHA were prepared in dimethylsulfoxide (DMSO, Sigma–Aldrich) and diluted to the tested final concentrations in the culture medium. The final concentration of DMSO did not exceed 0.04%.

### Colony Formation and Wound Healing Assay

Nasopharyngeal carcinoma cells were plated in six-well plates (1.0 × 10^3^ cells per well) and cultured for 7 days. The cells were then fixed with paraformaldehyde for 10 min and stained with 1% crystal violet for 5 min prior to the counting of colonies. For the wound healing assay, NPC cells (2 × 10^5^) were seeded in six-well plates. When confluency was reached, the cell monolayer was scraped with a 10-μl pipette tip. Cell migration was observed by microscopy 24 h later.

### Cell Apoptosis Assay

The apoptosis rate was evaluated using the annexin V-APC/7-AAD Apoptosis Detection kit (BD, 550474) according to the instructions from the manufacturer. Briefly, the cells were seeded into six-well tissue culture plates (4 × 10^5^ cells/well). Following treatment, the cells were collected, washed twice with cold phosphate-buffered saline (PBS), and resuspended in 500 μl 1X binding buffer. Then, 5 μl annexin V-APC and 5 μl 7-AAD were added to the buffer and incubated at room temperature for 15 min in the dark. The cells were analyzed by flow cytometry (BD Biosciences, San Jose, CA, United States) within 1 h.

### Western Blotting

Western blotting was performed as previously mentioned (Li et al., 2009). Briefly, NPC cells were lyzed as indicated in 0.3% Nonidet P40 (Sigma–Aldrich, 74388) buffer containing 150 mM NaCl, 50 mM Tris-HCl, pH = 7.5, and complete protease inhibitor cocktail (Roche, 04693132001). The following primary antibodies were commercially obtained: pan anti-acetyl-lysine (Kac) antibodies (PTM Biolab, 1:3,000 working dilution), H3 (Abcam, 18521; 1:1,000 working dilution), p-P53 Ser315 (Cell Signaling Technology, 2528; 1:1,000 working dilution), p-P53 ser37 (Cell Signaling Technology, 9289; 1:1,000 working dilution), P53 (Cell Signaling Technology, 9282; 1:1,000 working dilution), p-Rb1 ser807/811 (Cell Signaling Technology, 9308; 1:1,000 working dilution), Rb1 (Cell Signaling Technology, 9313; 1:1,000 working dilution), PARP 1 (Cell Signaling Technology, 9542; 1:1,000 working dilution), caspase 9 (Cell Signaling Technology, 52873; 1:1,000 working dilution), Bax (Cell Signaling Technology, 27745; 1:1,000 working dilution), Bcl2 (Cell Signaling Technology, 15071; 1:1,000 working dilution), ACTB (Sigma–Aldrich, A5441; 1:10,000 working dilution), ERK (Abcam, 17942; 1:1,000 working dilution), p-ERK Thr202/Tyr204 (Cell Signaling Technology, 9101; 1:1,000 working dilution), HER2 (Cell Signaling Technology, 2242; 1:1,000 working dilution), and p-HER2 Tyr1221/1222 (Cell Signaling Technology, 2249; 1:1,000 working dilution). ImageJ software (version 1.45s) was used to quantify the gray value of the western blot results. The western blot image was digitized to calculate the mean ± SD with Student’s *t*-test (*p* < 0.05).

### Histone Extraction

The core histones were extracted using a total histone extraction kit (The Epigentek Group Inc., OP-0006) according to the manufacturer’s instructions. Briefly, NPC cells were harvested and re-suspended in 1× pre-lysis buffer, and the cells were lyzed on ice for 10 min with gentle stirring. Then, whole-cell lysates were further diluted in three volumes of lysis buffer and incubated on ice for 30 min. After that, the samples were centrifuged at 12,000 r/min for 5 min at 4°C, and the supernatant fraction (containing acid-soluble proteins) was transferred into a new vial with 0.3 volumes of the balance-DTT buffer added immediately.

### Protein Extraction and Digestion

The cells were harvested, then washed with ice-cold PBS, and lyzed by incubation in SDS lysis buffer. After quantification of the total protein, protein digestion was performed according to the filter-aided sample preparation procedure. Briefly, 200 μg of proteins was reduced with 100 mM DTT, and then 200 μl UA buffer (8 M urea, 150 mM Tris-HCl, pH 8.0) was added. The mixture was then loaded into Microcon Ultracel YM-10 filtration device and centrifuged at 14,000 × *g* for 15 min. The concentrates were then diluted with 200 μl UA buffer and centrifuged at 14,000 × *g* for 15 min. After centrifugation, the concentrates were alkylated in 100 μl IAA (50 mM IAA in UA) for 30 min in the dark. After centrifugation for 10 min, the concentrates were washed twice with UA buffer and twice with 100 mM NH_4_HCO_3_. Subsequently, trypsin solution (8 μg trypsin in 40 μl NH_4_HCO_3_ buffer) was added to the filter, and the proteins were incubated at 37°C overnight. Tryptic peptides were collected by centrifugation, followed by an additional wash with an elution solution [70% acetonitrile (ACN) and 0.1% formic acid]. Finally, the peptide mixture was desalted with C18-SD Extraction Disk Cartridge, and the peptide concentration was assayed by measuring the absorbance at 280 nm.

### Tandem Mass Tagging Labeling

Experiments were performed for three biological replicates. For phosphoproteome, three technical replicates were applied as well. The dried peptides were redissolved in 0.5 M TEAB and processed according to the manufacturer’s protocol for a six-plex TMT kit (Thermo Fisher Scientific). Briefly, one unit of TMT reagent (used to label the tryptic peptides of 100 μg proteins) was thawed and reconstituted in 24 μl ACN. The peptide mixture was incubated with the prepared TMT reagent (1 h, room temperature) and then quenched by the addition of 200 μl of 5% hydroxylamine for 15 min. Next, the TMT-labeled peptide mixtures were pooled equally (1:1:1:1:1:1), desalted, and dried by vacuum centrifugation.

### Phosphopeptide Enrichment

Phosphopeptide enrichment was performed as described by Larsen et al. (2005). Briefly, lyophilized peptides were re-suspended in DHB buffer [3% w/v DHB, 80% v/v ACN, 0.1% v/v trifluoroacetic acid (TFA)]. Then, TiO_2_ beads (GL Sciences, Japan) were added, and the mixture was agitated for 40 min. TiO_2_ beads were recovered by centrifugation and washed three times with washing buffer I (30% ACN and 3% TFA) and three times with washing buffer II (80% ACN and 0.3% TFA). Lastly, the phosphopeptides were eluted with the elution buffer (5% NH_4_OH/50% ACN), followed by lyophilization and MS analysis.

### LC–ESI–MS/MS Analysis by Q-Extractive MS

Peptides were dissolved in solvent A (0.1% FA) and loaded onto a Thermo scientific EASY column (C18 column, 5 μm, 100 μm × 2 cm, Thermo Scientific). Peptide separation was performed using a reversed-phase analytical column (C18 column, 75 μm × 250 mm, 3 μm, Thermo Scientific). The gradient was comprised of an increase from 0 to 55% solvent B (0.1% FA in 98% ACN) for 220 min, 55 to 100% for 8 min, and then holding at 100% for the last 12 min, at a constant flow rate of 250 nl/min on an EASY-nLC 1000 UPLC system. The eluted peptides were analyzed by Q Exactive^TM^ hybrid quadrupole-Orbitrap mass spectrometer (Thermo Scientific). A data-dependent procedure was one MS scan (*m/z* range of 350–1,800) followed by 10 MS/MS scans for the top 20 precursor ions. Dynamic exclusion was enabled, with exclusion duration of 30 s. Automatic gain control was set as 3e6 to prevent overfilling of the ion trap. The peptides were detected in the Orbitrap at a resolution of 70,000. Peptides were selected for MS/MS using NCE setting as 29, and ion fragments were detected at a resolution of 17,500.

### MS Data Analysis

MS/MS spectra were searched using Mascot 2.2 (Matrix Science) embedded in Proteome Discoverer 1.4 against the UniProt human FASTA (released on 5/5/2018). For protein identification, the following options were used: peptide mass tolerance, 20 ppm; MS/MS tolerance, 0.1 Da; enzyme, trypsin; missed cleavage, 2; fixed modifications, carbamidomethyl (C); variable modifications, TMT 6-plex (N-term), TMT 6-plex (K), oxidation (M), phosphorylation (S/T/Y), and false discovery rate (FDR) ≤ 0.01. Proteome Discoverer 1.4 was used to extract the peak intensity of each expected TMT reporter ion from the fragmentation spectrum. Only spectra in which all quant channels are present were used for quantification. The score threshold for peptide identification was set at 1% FDR and with PhosphoRS site probability cutoff of 0.75. Student’s *t*-test was used to evaluate the statistical significance, and FDR was calculated. The criteria for significant abundance changes were abundance ratios ≥ 1.2 and *P*-value ≤ 0.05.

### Bioinformatic Analysis

Metascape, a gene annotation and analysis resource^1^, was employed to conduct gene enrichment and functional annotation analyses. The gene ontology (GO) annotation of DEPs was derived from the UniProt-GOA database^2^. DEPs were classified by GO annotation based on three categories, including biological processes (BPs), cellular compartments, and molecular functions (MFs), and *p* < 0.05 was considered statistically significant. Pathway enrichment analysis was performed by IPA software (Ingenuity Systems) and KEGG pathway database^3^. The adjusted *P*-values < 0.05 were considered statistically significant.

### Statistical Analysis

Two-tailed Student’s *t*-tests were used for all comparisons. All values included in the figures represent the mean values ± SD. Error bars represent ± SD for triplicate experiments. The statistical significance is indicated with asterisks (^∗^). A two-sided *P*-value < 0.05 was considered statistically significant (^∗^*P* < 0.05, ^∗∗^*P* < 0.01, ^∗∗∗^*P* < 0.001).

## Results

### SAHA Inhibits the Proliferation of NPC Cells

**FIGURE 1:**
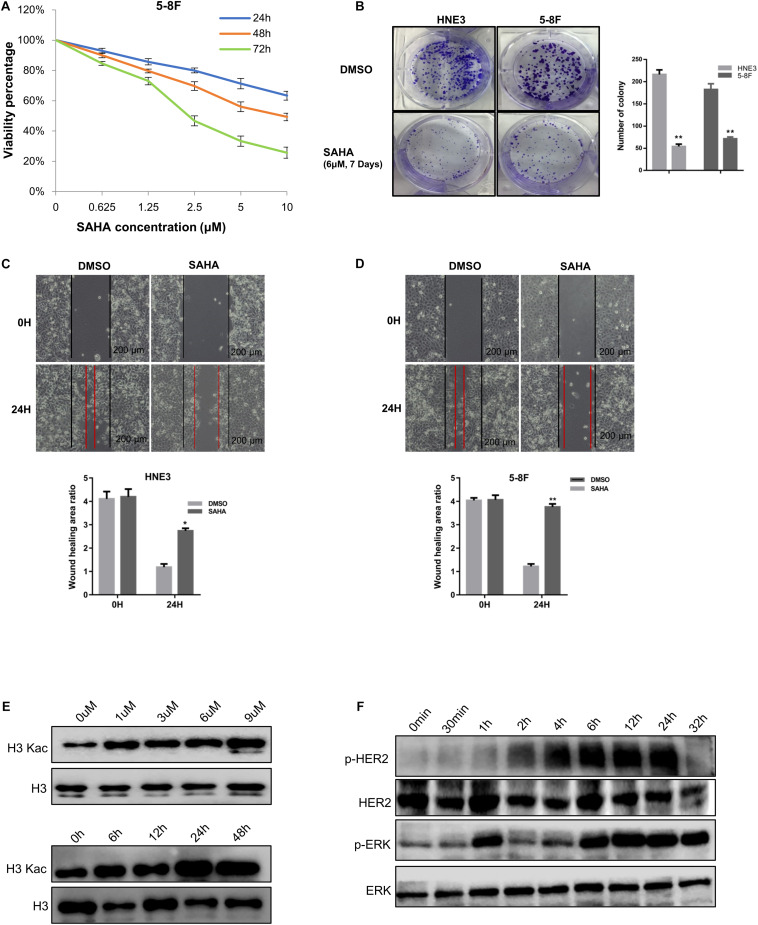
Suberoylanilide hydroxamic acid (SAHA) inhibits cell proliferation and migration in nasopharyngeal carcinoma (NPC) cells. **(A)** SAHA treatment suppressed NPC cell growth. 5-8F cells were treated with various concentrations of SAHA for 24, 48, and 72 h, respectively. **(B)** SAHA treatment inhibited the proliferation of NPC cells. HNE3 and 5-8F cells were treated with SAHA or dimethylsulfoxide (DMSO) as indicated and analyzed by a colony formation assay (left panel). A quantitative analysis of the colony was performed by ImageJ (right panel). **(C,D)** SAHA treatment inhibited the migration of NPC cells. HNE3 **(C)** and 5-8F cells **(D)** were treated with SAHA or DMSO as indicated and analyzed by wound healing assay. Scale bars: 200 μm. A quantitative analysis of the wound healing area was performed by ImageJ. **(E)** SAHA treatment affected the acetylation of histone H3 in NPC cells. 5-8F cells were treated with DMSO or SAHA at the indicated concentrations (upper panel) and time points (lower panel). **(F)** SAHA treatment affected protein phosphorylation in NPC cells. Immunoblots of whole-cell lysates from 5-8F cells treated with DMSO or SAHA at indicated time points were analyzed. Data are shown as mean ± SD (*n* = 3) or typical photographs of one representative experiment. Similar results were obtained in three independent experiments. *p < 0.05, **p < 0.01.

To investigate the effects of SAHA on NPC cell proliferation, NPC cells (5-8F, 6-10B, and HNE3) were treated with increasing concentrations of SAHA. SAHA treatment markedly decreased cell survival compared with the untreated controls in a dose- and time-dependent manner (Figure 1A and Supplementary Figure S1). Similarly, colony formations were robustly inhibited in HNE3 and 5-8F cells upon SAHA treatment (Figure 1B). Next, we performed wound healing assay and observed that SAHA treatment intensively suppressed cell migration in NPC cells (Figures 1C,D). As SAHA is a pan HDAC inhibitor, we examined the lysine acetylation level of histone H3 in NPC cells by western blotting. As shown in Figure 1E, a significant augmentation of H3Kac signals was detected as the concentration and the treatment time of SAHA increased. The maximum H3Kac signal was detected at 6 μM and 24 h of SAHA treatment. Since cell viability was still nearly 80% when treated at 6 μM for 24 h, this condition was applied for all the following experiments in NPC cells. Interestingly, we observed that SAHA altered HER2 and ERK phosphorylation in 5-8F cells utilizing the handy phosphorylated antibodies (Figure 1F), which gave us hints to investigate protein phosphorylation upon SAHA stimulation.

Taken together, these results showed that SAHA inhibited the proliferation and the migration of NPC cells. Meanwhile, protein phosphorylation was regulated by SAHA treatment.

### Profile of Phosphoproteomics Data Toward SAHA Treatment in NPC Cells

**FIGURE 2:**
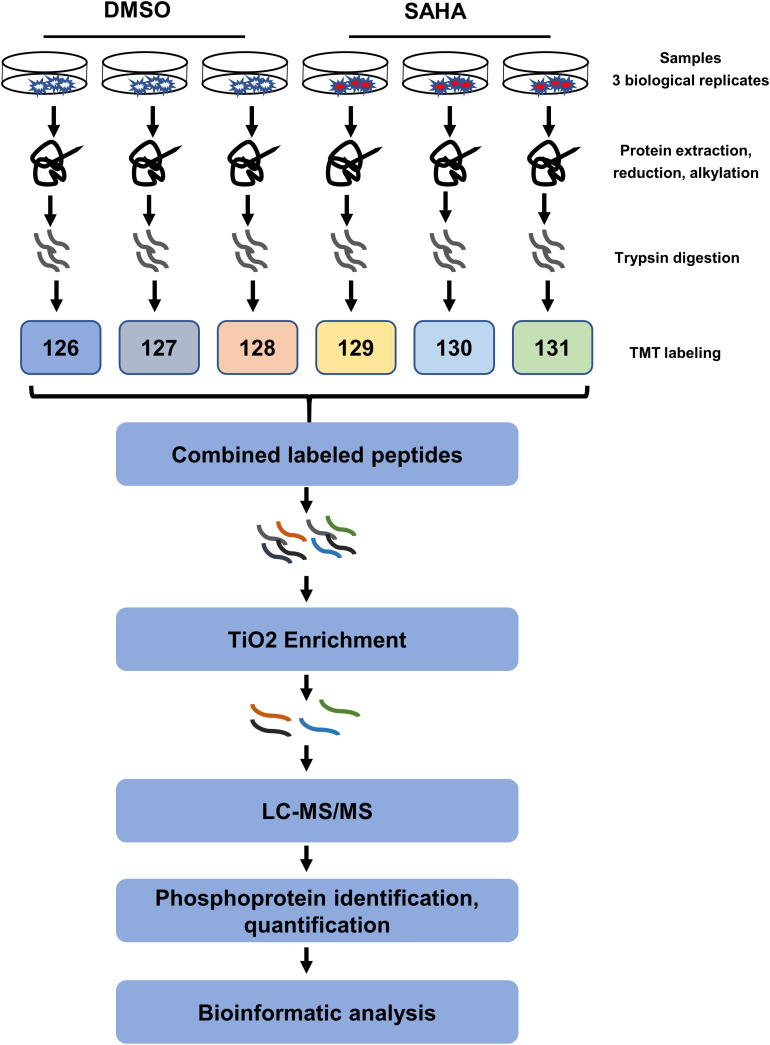
Schematic illustration of the (TMT)-based quantitative phosphoproteomic workflow. 5-8F cells treated with suberoylanilide hydroxamic acid for 24 h were subjected to six-plex TMT labeling. Combined labeled peptides were subjected to TiO_2_ enrichment. The enriched phosphopeptides were analyzed using an Orbitrap-equipped mass spectrometer. TMT, tandem mass tag; LC–MS/MS, liquid chromatography–tandem mass spectrometry; TiO_2_, titanium dioxide.

In order to better understand the anti-tumor mechanisms of SAHA in NPC cells, 5-8F cells were treated with SAHA or DMSO, and mass spectrometry was performed on whole-cell lysates with quantitative phosphoproteomic technology by six-plex TMT to detect alterations in protein phosphorylation. The workflow of TMT-based phosphoproteomic analysis is demonstrated in Figure 2.

**FIGURE 3:**
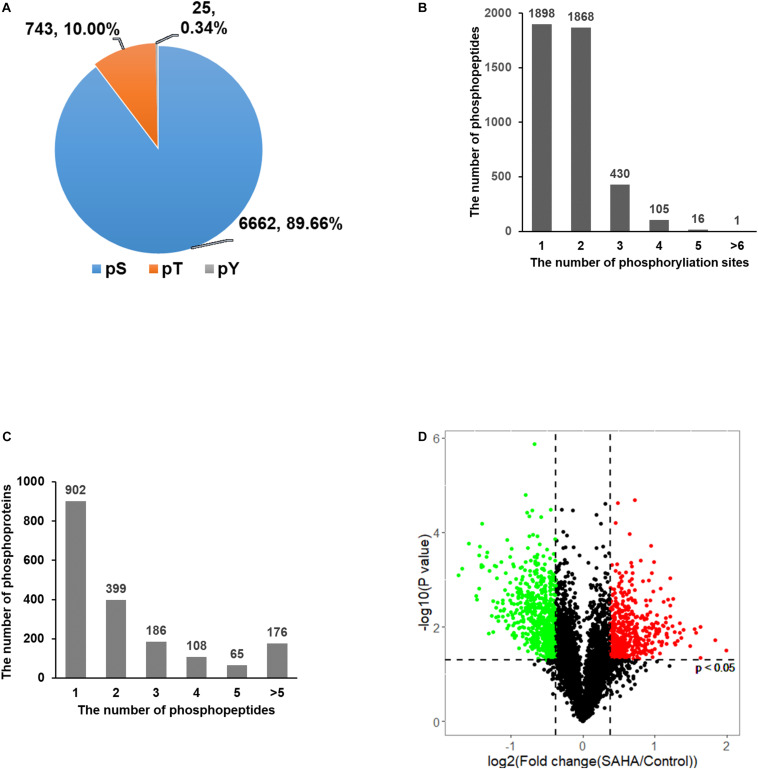
Distribution of phosphorylation sites. **(A)** The distribution of phosphorylation on serine, threonine, and tyrosine showed that phosphorylated serine was dominant. **(B)** The distribution of peptides with single- and multi-phosphorylation sites showed that the majority of phosphopeptides have only one or two phosphorylation sites. **(C)** The distribution of phosphoproteins based on the number of identified phosphopeptides showed that nearly half of the proteins have only one phosphopeptide. **(D)** Volcano plots of group comparisons [suberoylanilide hydroxamic acid (SAHA) *versus* dimethylsulfoxide] showing the adjusted significance *P*-value (log2) *versus* fold change (log2). The plots indicate the most robust protein changes in SAHA. The horizontal gray dotted lines indicate an adjusted *P*-value threshold of 0.05; the vertical gray dotted lines indicate a fold change threshold of 20%. Significantly upregulated phosphopeptides are marked in red and those downregulated are in green (adjusted *p*-value < 0.05).

Overall, 7,430 phosphorylation sites in 2,424 phosphoproteins were identified, of which 4,318 phosphorylation sites in 1,836 phosphoproteins were quantified (Supplementary Table 1). A total of 6,662 (89.66%) of the sites were found at serine, 743 (10%) at threonine, and 25 (0.34%) at tyrosine residues (Figure 3A). Among the quantified 4,318 phosphopeptides, 1,898 (43.96%) phosphopeptides contained single phosphorylation, 1,868 (43.26%) phosphopeptides had two phosphorylated sites, and 552 (12.78%) phosphopeptides were phosphorylated at more than two sites (Figure 3B). For proteins, 902 (49.13%) of 1,836 phosphoproteins contained a single phosphorylated site, 399 (21.73%) proteins harbored two phosphorylated sites, and 535 (29.14%) proteins have three or more than three phosphorylated sites (Figure 3C).

Among these phosphorylation sites, 2,098 showed a significant change upon SAHA stimulation (*p* < 0.05; fold change > 1.2 or < −1.2). A total of 847 phosphorylation sites were upregulated and 647 sites were downregulated upon SAHA treatment (Figure 3D).

### Identification of Differentially Expressed Phosphoproteins

**FIGURE 4:**
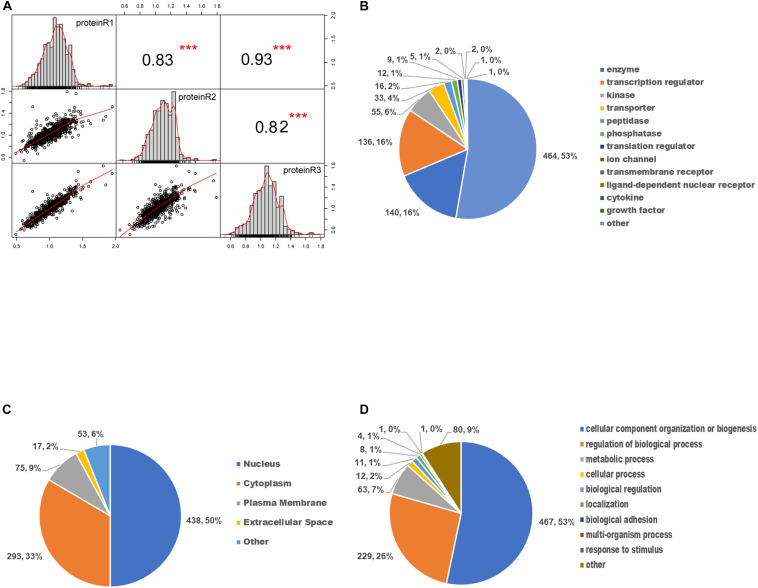
Gene ontology enrichment analysis of differentially expressed phosphoproteins. **(A)** The scatter plot matrix of Pearson correlation between replicate experiments (proteinR1-R3) shows that the reproducibility is high (***p < 0.001). **(B)** Molecular function analysis of differentially expressed phosphoproteins. **(C)** Cellular component analysis of differentially expressed phosphoproteins. **(D)** Biological process analysis of differentially expressed phosphoproteins.

We first evaluated the reproducibility of the quantification. Pearson correlation coefficient analysis for phosphopeptides based on the ratio was performed to assess the similarity between biological replicates (Figure 4A). A higher correlation between any two of the three replicates was observed with *R*^2^ value higher than or equal to 0.82, indicating that the results had reasonable technical and instrument variation.

To determine the involved protein categories, functions, and localizations of the DEPs upon SAHA treatment, the GO-based classification and enrichment analysis were conducted. As shown in Figure 4B, the MF category indicated that the DEPs are mainly associated with enzymes (464, 53%), peptidases (140, 16%), transcription regulators (136, 16%), and kinases (55, 6%). The cellular component category showed that DEPs are mainly involved in the nucleus (438, 50%), cytoplasm (293, 33%), plasma membrane (75, 9%), and extracellular space (17, 2%) (Figure 4C). In BP analysis, DEPs were markedly enriched in cellular component organization or biogenesis (467, 53%), regulation of BP (229, 26%), metabolic process (63, 7%), and cellular process (12, 2%) (Figure 4D).

### Functional Characteristics and IPA Pathway Analysis of DEPs

**FIGURE 5:**
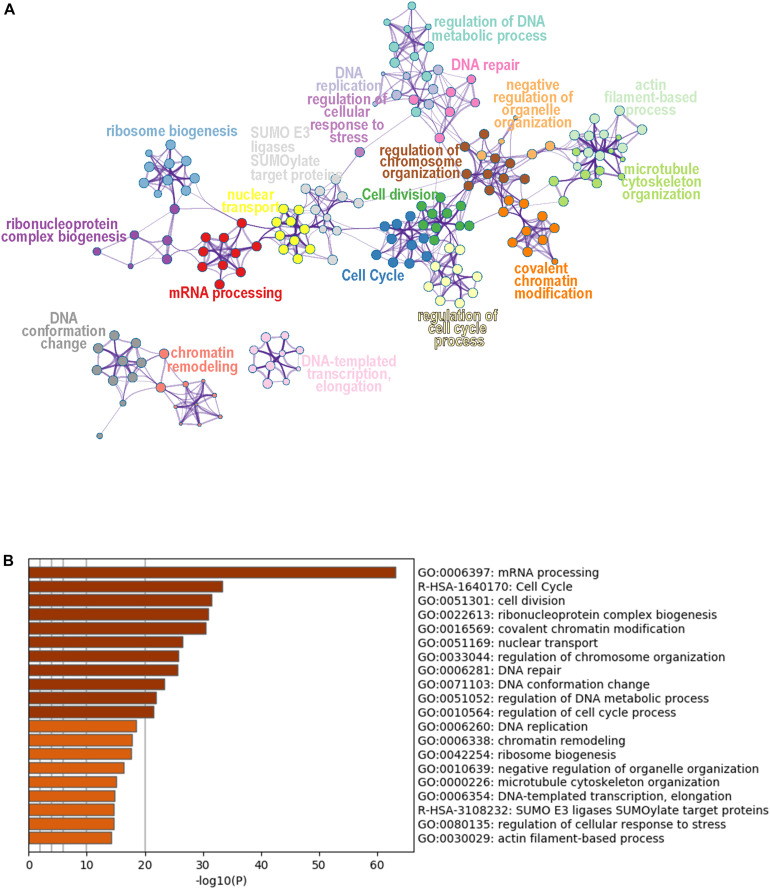
Cluster analysis of differentially expressed phosphoproteins. **(A)** Network plot of the relationships among enriched terms. Nodes representing enriched terms were grouped into clusters based on their membership similarities and colored by their cluster ID; nodes that share the same cluster ID are typically close to each other. **(B)** Bar graph of the top 20 enriched terms across input gene lists, colored by *p*-value.

To obtain a global view of the cellular functions of the DEPs toward SAHA treatment, we performed comprehensive bioinformatic analysis based on GO, KEGG pathway, and protein complex database (Figure 5). According to their membership similarities, enriched processes or pathways were grouped into different clusters. In Figure 5A, the top 20 enrichment processes or pathways are presented, with different notes referring to their corresponding enriched terms. The colors represented different cluster IDs, of which nodes of the same color indicated the similarity of their cellular functions. Then, to further study these enriched processes, we ranked them based on the enrichment degree (Figure 5B). The results showed that the processes or pathways associated with mRNA processing, cell cycle, cell division, ribonucleoprotein complex biogenesis, covalent chromatin modification, and nuclear transport were robustly enriched, which might serve as the potential targets of SAHA.

**FIGURE 6:**
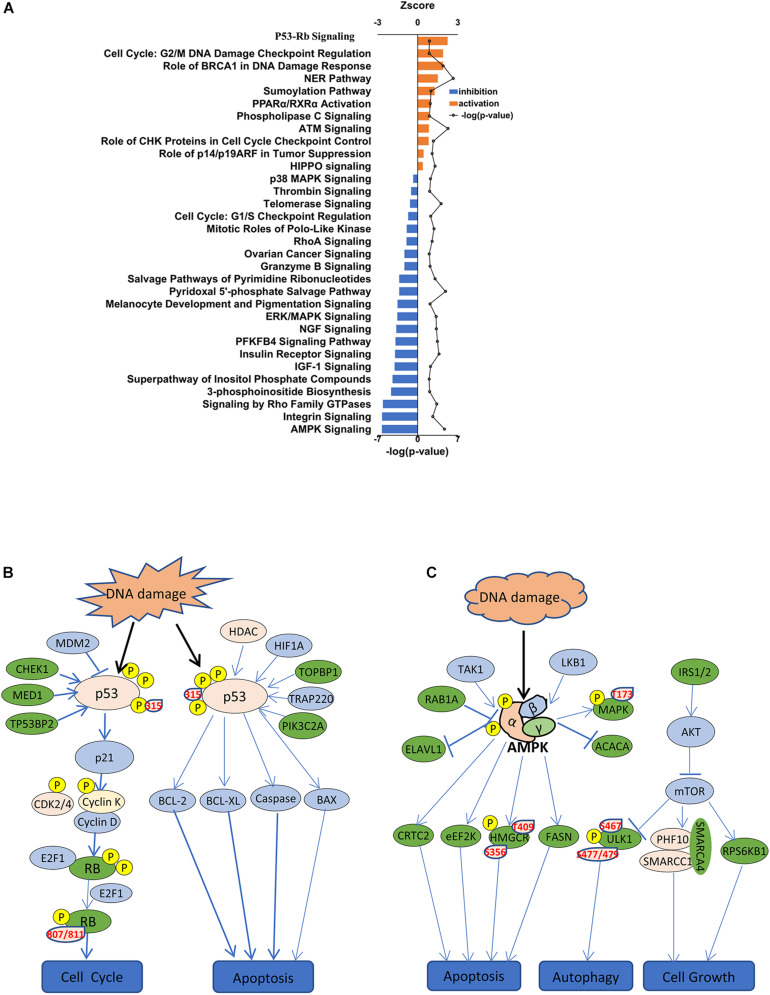
IPA signaling pathway analysis of differentially expressed phosphoproteins. **(A)** IPA showing decreased and increased biological functions of differentially expressed phosphoproteins in suberoylanilide hydroxamic acid-treated cells. Depicted are functions with an activation score (*z*-score) > 0 (increased activation) or < 0 (decreased activation). **(B)** Activated p53 signaling pathway is presented. Significantly upregulated phosphoproteins are shown in pink, while downregulated phosphoproteins are shown in green. **(C)** Inhibited AMPK signaling pathway is presented. Significantly upregulated phosphoproteins are shown in pink, while downregulated phosphoproteins are shown in green.

To identify cellular signaling associated with the anti-tumor effects of SAHA in NPC cells, IPA signaling pathway analysis was performed. Overall, the DEPs were involved in 32 significant cellular pathways (*p* < 0.05 and FDR < 0.05) (Figure 6A and Supplementary Table 3). A comprehensive analysis of these pathways revealed that DEPs were markedly enriched in five important tumor-related molecular network systems, including cell proliferation, cell metabolism, cell cycle, cell adhesion, and cell signal transduction (Table 1). Upon SAHA treatment, the upregulated DEPs were mainly enriched in cell cycle, cell apoptosis, and DNA repair related pathways, while the downregulated DEPs were mainly harbored in energy metabolism, signal transduction, and cell proliferation relevant pathways (Figure 6A).

**TABLE 1:** The functional categories of identified pathways *via* IPA.

Category	Pathway	z-Score
Cell proliferation		
	P53 signaling	2.236
	HIPPO signaling	0.378
	Role of p14/p19ARF in tumor suppression	0.447
	PPARA/RXRA activation	1
	Granzyme B signaling	–1
	ERK/MAPK pathway	–1.528
	NGF signaling	–1.604
	3-Phosphoinositide biosynthesis	–2
Cell cycle		
	Cell cycle: G2/M DNA damage checkpoint regulation	1.89
	ATM signaling	0.832
	Role of CHK proteins in cell cycle checkpoint control	0.816
	Role of BRCA1 in DNA damage response	1.89
	Cell cycle G1/S checkpoint regulation	–0.707
	P38 MAPK pathway	–0.333
	Mitotic roles of Polo-like kinases	–0.816
	Melanocyte development and pigmentation signaling	–1.508
	Integrin signaling	–2.668
Cell metabolism		
	AMPK signaling	–2.683
	NER pathway	1.5
	Salvage pathways of pyrimidine ribonucleotides	–1.387
	Pyridoxal 5′-phosphate salvage pathway	–1.387
	PFKFB4 signaling	–1.667
	Insulin receptor signaling	–1.698
	IGF-1 signaling	–1.732
	Superpathway of inositol phosphate compounds	–1.886
Cell adhesion		
	Thrombin signaling	–0.5
	Telomerase signaling	–0.577
	RhoA signaling	–0.832
	Ovarian cancer signaling	–1
	Signaling by Rho family GTPases	–2.6
Cell signal transduction		
	Sumoylation pathway	1.265
	Phospholipase C signaling	0.853

Among the activated pathways found in this study, p53 signaling pathway, G2/M DNA damage checkpoint regulation, and the role of BRCA1 in DNA damage response were the three most significantly activated (z-score > 2) in response to SAHA treatment (Figure 6A). Representative p53 signaling pathways that are involved in cell cycle, cell proliferation, cell apoptosis, and other critical cellular processes are among the most robustly activated pathways (Figure 6B). In this study, 10 DEPs (CCNK, CDK2, CHEK1, CSNK1D, HDAC1, MED1, PIK3C2A, RB1, TOPBP1, and TP53) were identified in p53 pathway (Table 2 and Supplementary Table 4). On the other hand, the pathways associated with energy metabolism, including AMPK pathway, IGF-1 pathway, insulin receptor pathway, and PFKFB4 signaling pathway, were inhibited by SAHA, with AMPK pathway being the most inhibited one. A total of 12 DEPs (PPM1H, PPP2R5E, PRKAA1, PRKACA, PRKAR1A, PRKAR1B, RAB1A, RAB8A, RPS6KB1, SMARCA4, SMARCC1, and ULK1) involved in AMPK pathway were discovered *via* IPA signaling analysis (Table 3 and Supplementary Table 5), with several representative proteins presented in Figure 6C. The raw data of p53 signaling and AMPK signaling pathway from IPA database, along with information on all the identified proteins, are summarized in Supplementary Tables 4, 5. Collectively, these results revealed that SAHA manipulated the activity of critical molecules *via* phosphorylation to activate P53 and inactivate AMPK pathways, leading to the proliferation arrest and apoptosis of NPC cells.

**TABLE 2:** Description of identified molecules in the P53 pathway.

Accession	Symbol	Description	Phosphorylation sites	*p*-Value
G3V5E1	CCNK	Cyclin K	S340	0.0322
G3V317	CDK2	Cyclin-dependent kinase 2	Y15	0.000411
J3KN87	CHEK1	Checkpoint kinase 1	S331	0.0296
B4DIJ9	CSNK1D	Casein kinase 1 delta ase	T236	0.0132
Q5HYD4	HDAC1	Histone diacetyl 1	S421/S423	0.00595
B2RAG9	MED1	Mediator complex subunit 1	T1051/T1057	0.0144
A0A0C4DGF9	PIK3C2A	Phosphatidylinositol-4-phosphate3-kinase catalytic subunit type2 alpha	S259	0.0338
P06400	RB1	RB transcriptional corepressor 1	S807/S811, T821/T826, S788/S795, S37, T821/T826, S612, S249/T252	0.0254
A0AV47	TOPBP1	DNA topoisomerase II binding protein 1	T774/S777	0.0141
I3L0W9	TP53	Tumor protein p53	S315	0.00857
B4DI25	TP53BP2	Tumor protein p53 binding protein 2	S183/S196	0.0163

**TABLE 3:** Description of identified molecules in the AMPK pathway.

Accession	Symbol	Description	Phosphorylation sites	*p*-Value
Q13085	ACACA	Acetyl-CoA carboxylase alpha	S2343	0.00832
Q4LE49	ARID1A	AT-rich interaction domain 1A	S418/S426	0.000503
Q5T4K5	CRTC2	CREB-regulated transcription coactivator 2	S136/T138	0.0284
O00418	EEF2K	Eukaryotic elongation factor 2 kinase	S71	0.0113
Q15717	ELAVL1	ELAV-like RNA binding protein 1	S202	0.00685
A0A0U1RQF0	FASN	Fatty acid synthase	S207	0.00722
A8KA27	HMGCR	3-Hydroxy-3-methylglutaryl-CoA reductase	T409, S356	0.0154
P35568	IRS1	Insulin receptor substrate 1	S629/S636	0.0034
Q96RG4	IRS2	Insulin receptor substrate 2	S736/S737	0.0212
J3QL77	MAP2K3	Mitogen-activated protein kinase 3	S3/S15	0.0357
Q9UG54	MAP3K7	Mitogen-activated protein kinase 7	S93	0.0485
B4DHN0	MAPK1	Mitogen-activated protein kinase 1	T173	0.0178
B4DK20	PHF10	PHD finger protein10	S115/S119	0.00465
A0A0C4DGF9	PIK3C2A	Phosphatidylinositol-4-phosphate 3-kinase catalytic subunit type 2 alpha	S259	0.0338
Q9ULR3	PPM1H	Protein phosphatase, Mg_2_ + Mn_2_ + dependent 1H	S221/S223	0.0188
Q86XZ2	PPP2R5E	Protein phosphatase 2 regulatory subunit B’epsilon	S33/S34	0.0465
Q13131	PRKAA1	Protein kinase AMP-activated catalytic subunit alpha 1	S486/T490	0.0259
Q15136	PRKACA	Protein kinase cAMP-activated catalytic subunit alpha	T179	0.00915
K7EMU2	PRKAR1A	Protein kinase cAMP-activated catalytic subunit alpha	S83	0.00108
C9J4C2	PRKAR1B	Protein kinase cAMP-dependent type I regulatory subunit beta	S77/S83, S71/S77/S83	0.00282
Q96RD8	RAB1A	RAB1A, member RAS oncogene family	T84	0.0223
P61006	RAB8A	RAB8A, member RAS oncogene family	S181/S185	0.00109
B4DDM0	RPS6KB1	Ribosomal protein S6 kinase B1	S403/S408, S397/T400/S403/S408	0.00092
P51532	SMARCA4	SWISNF-related, matrix-associated, actin-dependent regulator of chromatin, subfamily a, member 4	S613, S1417/T1423, T1423 S695/S699	0.00768
Q05BW5	SMARCC1	SWISNF-related, matrix-associated, actin-dependent regulator of chromatin subfamily c member 1	S328/S330, S310	0.00936
O75385	ULK1	unc-51 like autophagy activating kinase 1	S467, S477/S479	0.0137

### Validating the Activation of p53–Rb1 Axis by SAHA Treatment in NPC Cells

**FIGURE 7:**
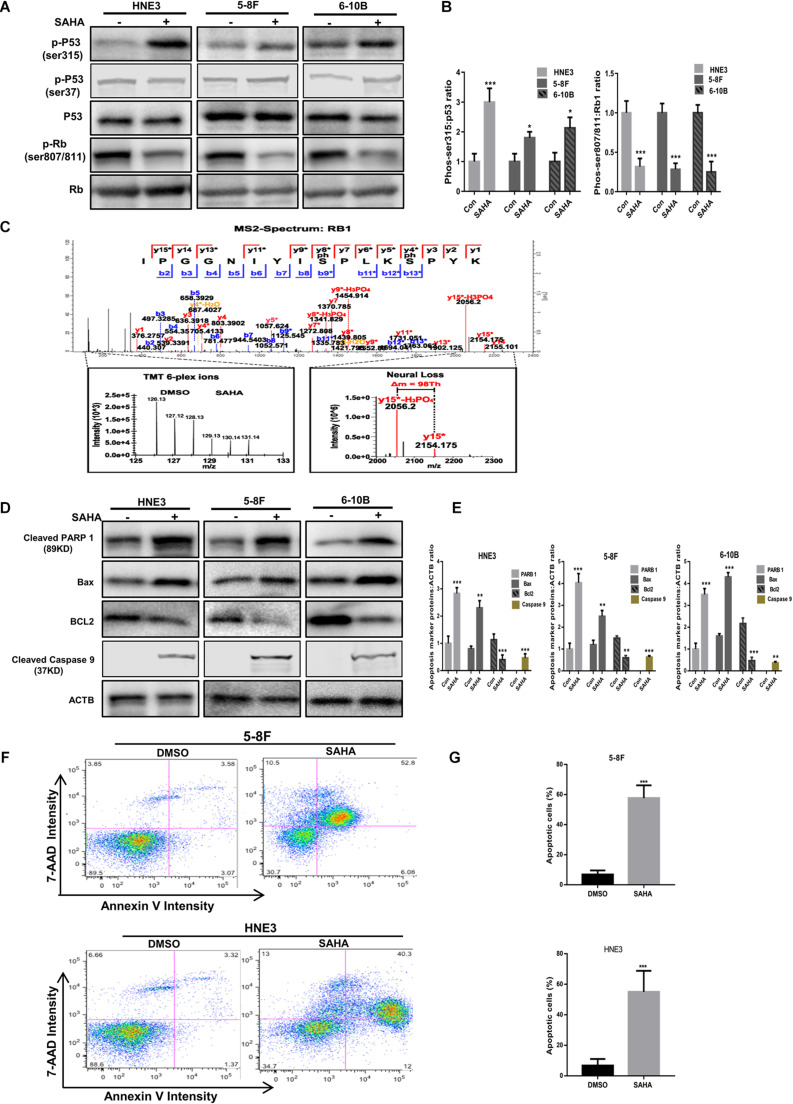
Validation of the activation of p53 pathway and apoptosis by suberoylanilide hydroxamic acid (SAHA). **(A**,**B)** Immunoblot analysis of p53 and Rb1 phosphorylation in nasopharyngeal carcinoma (NPC) cells with or without SAHA treatment. The whole-cell lysates extracted from HNE3, 5-8F, and 6-10B were subjected to Western blot analysis with the indicated antibodies. The protein levels of p53 and Rb1 are shown in **(A)**, while the statistical analysis of all samples is shown in **(B)**. The single asterisk denotes *P* < 0.05. The triple asterisks denote *P* < 0.001. Error bars represent ± SD of triplicate experiments. **(C)** Mapping and quantification of Rb1 phosphopeptide. Representative MS2 spectrum corresponding to a tryptic peptide derived from Rb1 containing two serine phosphorylation sites is presented. **(D,E)** Immunoblot analysis of cleaved PARP 1, cleaved caspase 9, Bax, and Bcl2 in NPC cells with or without SAHA treatment. The whole-cell lysates extracted from HNE3, 5-8F, and 6-10B were subjected to Western blot analysis with the indicated antibodies. The protein levels of cleaved PARP 1, cleaved caspase 9, Bax, and Bcl2 are shown in **(D)**, while the statistical analysis of all samples is shown in **(E)**. The single asterisk denotes *P* < 0.05; the double asterisks denote *P* < 0.01. The triple asterisks denote *P* < 0.001. Error bars represent ± SD of triplicate experiments. **(F,G)** Apoptosis was measured by annexin-V staining in NPC cells. HNE3 and 5-8F were treated with dimethylsulfoxide or SAHA for 24 h and stained with annexin-V-APC and 7-AAD **(F)**. The percentage of apoptotic cells is shown in **(G)**. The triple asterisks denote *P* < 0.001. Error bars represent ± SD of triplicate experiments.

As presented in Figure 6B, our quantitative phosphoproteomic data indicated that p53 phosphorylation at Ser315 residue was increased upon SAHA treatment in NPC cells. To validate this finding, we assessed the phosphorylation level of p53 at Ser315 site as well as at Ser37 site by western blotting analysis. As shown in Figures 7A,B, the phosphorylation signals of Ser315 residue, but not Ser37 residue, were significantly elevated by SAHA treatment in different NPC cell lines, including 5-8F, HNE3, and 6-10B cells (*P* < 0.001).

Rb1 is a well-known tumor suppressor gene, the dephosphorylated type of which binds E2F and blocks its ability to activate transcription. Here we observed that several sites (S807/S811, T821/T826, S788/S795, S37, T821/T826, S612, and S249/T252) were downregulated on the phosphorylation level of Rb1 upon SAHA treatment, of which S807/S811 is the most downregulated (Table 2). A representative MS2 spectrum of Rb1 phosphorylated peptide containing two serine phosphorylation sites is presented (Figure 7C). Consistent with the quantitative phosphoproteomic analysis, the immunoblot results demonstrated that the phosphorylation signals of S807/S811 in Rb1 were reduced in SAHA-treated cells (Figures 7A,B). These results suggested that SAHA may exert its anti-tumor effect by regulating the phosphorylation of p53 and Rb1 at specific residues.

Since cell apoptosis-related pathways were markedly enriched upon SAHA treatment, we next determined the expression of PARP, Bax, and Bcl2 in NPC cells with or without SAHA treatment by western blotting. Compared with DMSO-treated cells, the expression levels of cleaved PARP 1, cleaved caspase 9, and Bax, but not Bcl2, were significantly increased by SAHA treatment (Figure 7D). The quantitative analyses of these proteins normalized to ACTB are shown in Figure 7E. This potentiation of apoptosis was further confirmed by flow cytometry analysis (Figures 7F,G). These observations demonstrated that SAHA treatment promoted cell apoptosis in NPC cells.

Taken together, our data indicated that SAHA activates tumor suppressors like p53 and Rb1 *via* phosphorylation and promotes cell apoptosis in NPC cells to implement its anti-tumor role.

## Discussion

Nasopharyngeal carcinoma is a highly malignant head and neck tumor. Despite that significant efforts have been put into investigation, early diagnostic biomarkers and efficient therapies are lacking (Cao et al., 2011), bringing a huge burden to the public health community and urging for novel therapies for NPC patients. Here we found that SAHA, a clinically approved drug, inhibited the proliferation and promoted the apoptosis of NPC cells. Applying quantitative phosphoproteomic analysis, we reported for the first time the phosphoproteomic profile and deciphered the potential anti-tumor mechanism of SAHA in NPC cells. Overall, our findings provided novel insights into the anti-tumor effect of SAHA in NPC cells, which may lead to new therapies for NPC patients.

As the most promising deacetylation inhibitor, SAHA has exhibited potent anti-tumor properties both in solid tumors and hematologic malignances *via* diverse mechanisms (Minucci and Pelicci, 2006; Siegel et al., 2009). In this study, we applied TMT-based quantitative proteomics associated with TiO_2_-based phosphor-peptide enrichment methodology to evaluate protein phosphorylation modulated during SAHA treatment in NPC cells. Our results identified 7,430 phosphorylation sites and 1,836 DEPs, including 847 significantly upregulated and 647 significantly downregulated phosphorylation sites, in response to SAHA stimulation. Several cancer-associated BPs and signaling pathways were enriched, including p53 signaling pathway, cell cycle, DNA damage response, cell metabolism, and cell proliferation *via* GO and IPA pathway analysis. It suggested that SAHA may modulate these BPs and signaling pathways to exert its anti-tumor effect in NPC cells. In agreement with our results, other groups demonstrated that HDAC inhibitors established an anti-tumor activity in head and neck cancer. Francesca et al. revealed that SAHA modulated EGFR receptor expression and reversed epithelial–mesenchymal transition in SCCHN cell lines to display synergistic anti-tumor effects in combination with gefitinib (Bruzzese et al., 2011). It also has reported that TSA suppressed cell migration and invasion through the downregulation of EGFR-Arf1 signaling in SCCHN (He et al., 2019). Moreover, some HDAC inhibitors are in advanced clinical trials either as alone or in combination with other conventional treatments (Han et al., 2015; Caponigro et al., 2016).

Among the upregulated pathways found in this study, p53 signaling pathway ranked top upon SAHA treatment. A comprehensive pathway analysis using IPA database uncovered that p53 phosphorylation at Ser315 residue was elevated upon SAHA treatment, as validated by western blotting. PTMs, including phosphorylation, acetylation, methylation, and sumoylation, regulate the stability and the activity of p53 in response to many stress signals (Luo et al., 2000; Barlev et al., 2001; Buschmann et al., 2001). Phosphorylation at different sites of p53 can have an inhibitory or a stimulatory role in modulating p53-dependent transcription (Bond et al., 1999; Unger et al., 1999; Hirao et al., 2000). Structurally, Ser315 residue localizes in the NLS sequence in the C-terminal region of p53, which contains a tetramerization domain and a regulatory domain (Hupp et al., 1995). Mechanistically, several studies indicated that phosphorylation at Ser315 stimulates its DNA binding function. Jeremy et al. demonstrated that phosphorylation at the Ser315 residue of p53 enhanced p53-dependent transcription through the cdk/cdc-dependent pathway (Blaydes et al., 2001). Additionally, James et al. showed that phosphorylation of p53 at Ser315 site significantly improved the sequence-specific DNA binding activity of p53 protein *in vitro*, possibly by cooperating with other modifications within the C-terminal negative regulatory domain or by alternating N-terminal modifications (Bischoff et al., 1990). Consistent with these findings, our study demonstrated that SAHA boosted the phosphorylation of Ser315 of p53 in NPC cells. However, the upstream signal governing the phosphorylation of Ser315 that is induced by SAHA remains elusive, calling for further investigation.

Due to the crucial suppressive role of p53 protein in the tumorigenesis, we further analyzed the downstream effectors in p53 signaling pathway and found that the phosphorylation of Rb1 at Ser807/811 residues was reduced by SAHA treatment. The retinoblastoma susceptibility gene (Rb1), as the first tumor repressor gene discovered, was described over 30 years ago to have a critical effect on many cellular processes, including cell proliferation, apoptosis, cell cycle, cell differentiation, and DNA repair (Friend et al., 1986; Dowdy et al., 1993; Classon and Harlow, 2002; Dyson, 2016). Phosphorylation and dephosphorylation are well-established mechanisms to regulate Rb1 functions. Dephosphorylated Rb1, an active state, binds and blocks E2F-mediated transcriptional activation. Conversely, Rb1 phosphorylation inactivates its repressive function (Krek et al., 1993; Burke et al., 2010). Ser807/811 locates in the C-terminal of Rb1,with its phosphorylation affecting multiple functions of Rb1, such as Rb1-E2Fs interaction, apoptosis, differentiation, chromosomal stability, and senescence (Ho and Dowdy, 2002; Burkhart and Sage, 2008). Kinases including cdk2/cdk3/cdk5/cdk9 and p38 have been identified to be responsible for Rb1 phosphorylation at Ser807/811 residues. In addition, phosphorylation at Ser807/811 induces structural changes of Rb1, thus impacting phosphorylation at other sites (Brown et al., 1999; Kõivomägi et al., 2011). In addition to these two sites, phosphorylation at many other sites has also been detected according to our quantitative phosphoproteomic data. To obtain a deeper insight on the anti-tumor activity of SAHA in NPC cells, phosphorylation at other sites of Rb1 and the functional outcomes will be investigated in the future.

Nasopharyngeal carcinoma is sensitive to radiotherapy, but the significant rate of relapse and distant metastasis after therapy is still a major cause for NPC lethality (Chang and Adami, 2006). Deep insights and novel therapies are desperately demanded to improve NPC patient prognosis. Chemotherapy combined with immunotherapy or chemotherapy alone demonstrated promising efficacy in several clinical trials for advanced or recurrent NPC (Chen et al., 2018; Ma et al., 2018). Our findings showed that SAHA alone or in combination with current strategies, including radiotherapy, chemotherapy, and immunotherapy, may serve as a potent candidate to combat NPC.

## Conclusion

In summary, we demonstrated that SAHA inhibits cell proliferation and promotes apoptosis in NPC cells. Taking advantages of TMT labeling, TiO_2_ enrichment, and high-resolution LC–MS/MS, we presented the comprehensive quantitative phosphoproteomic profile in NPC cells in response to SAHA treatment, providing a precious resource for further studies on SAHA and NPC. Our results represent a deeper understanding of the underlying mechanism of SAHA’s anti-tumor roles. Furthermore, our study indicated that SAHA may serve as a novel therapy for NPC patients.

## Data Availability Statement

The mass spectrometry proteomics data have been deposited to the ProteomeXchange Consortium (http://proteomecentral.proteomexchange.org) via the iProX partner repository with the dataset identifier PXD021029 (http://proteomecentral.proteomexchange.org/cgi/GetDataset?ID=PXD021029).

## Author Contributions

HH, YFu, MYL, and YC designed the study and conceived the experiments. HH and MYL performed the experiments. YZ, FP, YFe, and YFu provided technical and material support. HH, MLL, LC, and ZC analyzed and interpreted the data. HH and MYL wrote the manuscript. All the authors read and approved the final manuscript.

## Conflict of Interest

The authors declare that the research was conducted in the absence of any commercial or financial relationships that could be construed as a potential conflict of interest.

## Footnotes

1 http://metascape.org

2 http://www.ebi.ac.uk/GOA/

3 http://kobas.cbi.pku.edu.cn/

## Abbreviations

CCK-8: Cell Counting Kit 8
CTCL: cutaneous T cell lymphomas
DEPs: differentially expressed phosphoproteins
EBV: Epstein–Barr virus
FDA: Food and Drug Administration
HDACs: histone deacetylases
IMAC: immobilized metal affinity chromatography
LC–MS/MS: liquid chromatography–tandem mass spectrometry
NPC: nasopharyngeal carcinoma
PTMs: protein posttranslational modifications
SAHA: suberoylanilide hydroxamic acid
TiO_2_: titanium dioxide.
