# Supplementary material for: Dissection of Anti-tumor Activity of Histone Deacetylase Inhibitor SAHA in Nasopharyngeal Carcinoma Cells via Quantitative Phosphoproteomics
Source: Front Cell Dev Biol. 2020 Nov 26;8:577784. doi: 10.3389/fcell.2020.577784 (PMC7726116; doi:10.3389/fcell.2020.577784)
Supplement: Supplementary file 6 [file Table_1.docx]

## SUPPLEMENTAL INFORMATION

**Dissection of anti-tumor activity of histone deacetylase inhibitor SAHA in nasopharyngeal carcinoma cells via quantitative phosphoproteomics**

**Huichao Huang^1^, Ying Fu^1^, Ye Zhang^1^, Fang Peng^1^, Miaolong Lu^1^, Yilu Feng^1^, Lin Chen^2^, Zhuchu Chen^1,3^, Maoyu Li^1,3*^, Yongheng Chen^1,4^**^*^

^1^Department of Oncology, NHC Key Laboratory of Cancer Proteomics, XiangYa

Hospital, Central South University, Changsha 410008, China

^2^Molecular and Computational Biology Program, Departments of Biological Sciences and Chemistry, University of Southern California, Los Angeles, California 90089

^3^Department of Gastroenterology, Xiangya Hospital, Central South University, Changsha 410008, Hunan, China

^4^National Clinical Research Center for Geriatric Disorders, XiangYa Hospital, Central South University, Changsha 410008, Hunan, China

**Inventory of Supplemental Information**

**Figure S1: Linked to Figure 1**

**A**


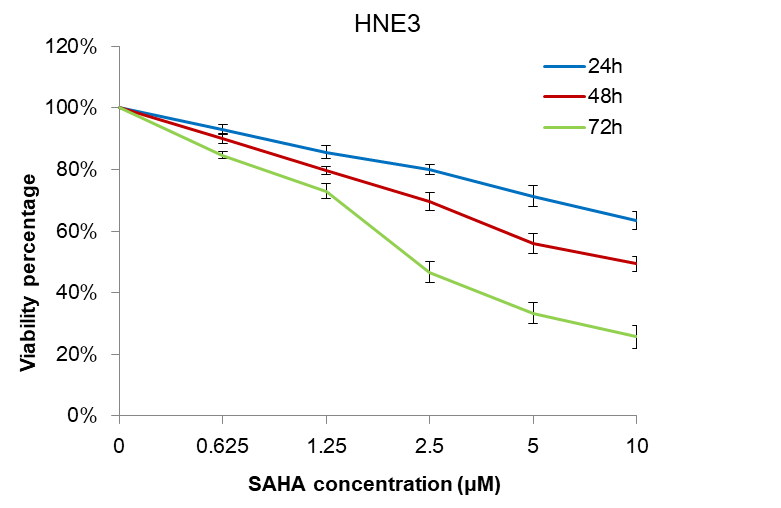


**B**


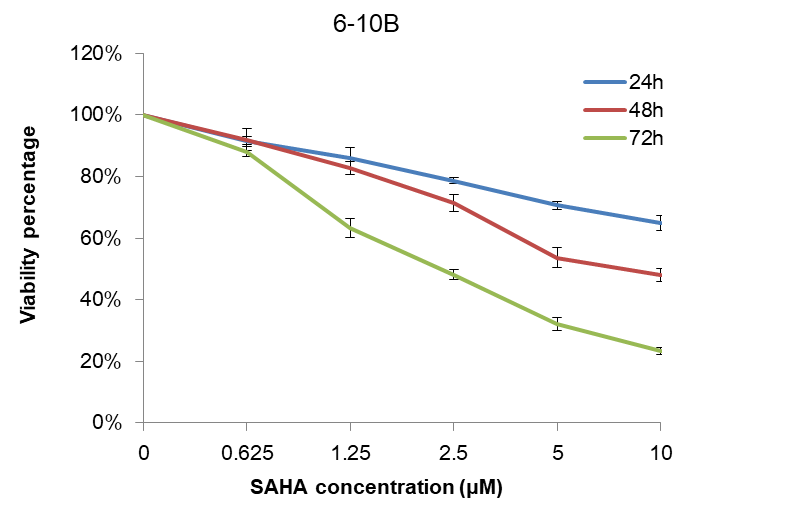


**Supplemental Figure 1. SAHA inhibits cell proliferation in NPC cells.** (A and B) SAHA treatment suppressed NPC cells growth. HNE3 cells (A) and 6-10B (B) were treated with various concentrations of SAHA for 24h, 48h, and 72 h, respectively.
